# Nitrifying Microbes in the Rhizosphere of Perennial Grasses Are Modified by Biological Nitrification Inhibition

**DOI:** 10.3390/microorganisms8111687

**Published:** 2020-10-29

**Authors:** Yi Zhou, Christopher J. Lambrides, Jishun Li, Qili Xu, Ruey Toh, Shenzhong Tian, Peizhi Yang, Hetong Yang, Maarten Ryder, Matthew D. Denton

**Affiliations:** 1 China-Australia Joint Laboratory for Soil Ecological Health and Remediation, Ecology Institute, Qilu University of Technology (Shandong Academy of Sciences), Jinan 250103, China; yi.zhou@adelaide.edu.au (Y.Z.); ruey.toh@adelaide.edu.au (R.T.); yanght@sdas.org (H.Y.); maarten.ryder@adelaide.edu.au (M.R.); matthew.denton@adelaide.edu.au (M.D.D.); 2 School of Agriculture, Food and Wine, The University of Adelaide, Urrbrae, SA 5064, Australia; xuqili1997@gmail.com; 3 School of Agriculture and Food Sciences, The University of Queensland, Brisbane, QLD 4072, Australia; chris.lambrides@uq.edu.au; 4 Institute of Agricultural Resources and Environment, Shandong Academy of Agricultural Sciences, Jinan 250013, China; tiansz1616@163.com; 5 College of Grassland Agriculture, Northwest A&F University, Yangling, Xianyang 712100, China; yangpeizhi@126.com

**Keywords:** microbial community, metagenomics, root exudation, turfgrass, forage

## Abstract

Soil nitrification (microbial oxidation of ammonium to nitrate) can lead to nitrogen leaching and environmental pollution. A number of plant species are able to suppress soil nitrifiers by exuding inhibitors from roots, a process called biological nitrification inhibition (BNI). However, the BNI activity of perennial grasses in the nutrient-poor soils of Australia and the effects of BNI activity on nitrifying microbes in the rhizosphere microbiome have not been well studied. Here we evaluated the BNI capacity of bermudagrass (*Cynodon dactylon* L.), St. Augustinegrass (*Stenotaphrum secundatum* (Walt.) Kuntze), saltwater couch (*Sporobolus virginicus*), seashore paspalum (*Paspalum vaginatum* Swartz.), and kikuyu grass (*Pennisetum clandestinum*) compared with the known positive control, koronivia grass (*Brachiaria humidicola*). The microbial communities were analysed by sequencing 16S rRNA genes. St. Augustinegrass and bermudagrass showed high BNI activity, about 80 to 90% of koronivia grass. All the three grasses with stronger BNI capacities suppressed the populations of *Nitrospira* in the rhizosphere, a bacteria genus with a nitrite-oxidizing function, but not all of the potential ammonia-oxidizing archaea. The rhizosphere of saltwater couch and seashore paspalum exerted a weak recruitment effect on the soil microbiome. Our results demonstrate that BNI activity of perennial grasses played a vital role in modulating nitrification-associated microbial populations.

## 1. Introduction

The application of N fertiliser is the most important nutritional component of the modern agricultural system following the Green Revolution but can lead to large environmental costs [1]. Unfortunately, 50 to 70% of the applied N is commonly lost, mainly through N leaching from nitrification [2]. Nitrification is the microbial oxidation of the relatively immobile ammonium-N (NH_4_^+^) to highly mobile nitrate (NO_3_^−^) via NH_4_^+^ → NH_2_OH → NO_2_^−^ → NO_3_^−^ which increases N leaching of NO_3_^−^ in soils [3].

Some plants are able to suppress soil nitrifiers by exuding secondary metabolites from roots, a process called biological nitrification inhibition (BNI) [3]. After the initial discovery in koronivia grass (*Brachiaria humidicola*) [4], BNI activity has been widely found in other forage grasses (e.g., *Brachiaria decumbens* [5] and *Hyparrhenia diplandra* [6]), major cereal crops (e.g., sorghum (*Sorghum bicolor*) [7] and rice (*Oryza sativa*) [8]), and agricultural weed species such as *Lolium rigidum*, *Bromus driandrus*, *Raphinus raphinastrum*, and *Avena fatua* [9]. In addition, genotypic variation in BNI within the same plant species has also been detected in wheat landraces and cultivars [10], and *B. humidicola* populations [11].

Plants with BNI functions originate from and/or are adapted to N-limited ecosystems—e.g., the BNI of koronivia grass was observed in Colombian low-N soil [4] and native grasses with BNI were found in low-nutrient savanna soils of West Africa [12]. Australia is the world’s driest inhabited continent and soils tend to be nutrient-impoverished [13]. Previous findings have revealed that the Australian native dicotylendous species *Hibiscus splendens* and *Solanum echinatum* showed a BNI capacity as strong as sorghum [14]. Australia has the highest proportional area covered by C4 grasses of all continents [15]. Naturalised perennial grass species are widely distributed and adapted in Australia, and potentially may exhibit BNI activity; however, screening and evaluation for BNI have not been extensively conducted.

Plant root exudation plays a key role in shaping the microbial community of the rhizosphere (the region closely surrounding plant roots in soil). Root exudates include diverse plant metabolites, and account for 10 to 50% of carbon fixed by photosynthesis [16]. Large-scale profiling of root metabolites and microbial genomes has demonstrated that the rhizosphere microbiome is structured by interactions between root exudate composition and microbial metabolite preferences [17]. Furthermore, knocking out specific plant genes controlling one or more groups of root exudate compounds such as benzoxazinoids [18], rosmarinic acid [19], and anti-fungal avenacins [20] significantly altered the assemblage of the rhizosphere microbiome, compared with the wild type. In plants capable of BNI, biological nitrification inhibitors from root exudations have been characterised as sorgoleone in sorghum [21], brachialactone in koronivia grass [22], and 1,9-decanediol in rice [8]. Sorgoleone was reported to reduce populations of ammonia-oxidizing archaea, which in turn limited ammonia oxidation [23]. As there are strong co-occurrence interactions between different microbial species in the rhizosphere [24], modifications to populations of ammonia-oxidizing-related microbes by biological nitrification inhibitors secreted from roots might affect the structure of the entire bacterial and archaeal communities and the abundance of other taxa in the rhizosphere; however, this possibility has not been studied to date.

Here, we used C4 perennial grass species native or naturalised in Australia, including bermudagrass (*Cynodon dactylon* L.), St. Augustinegrass (*Stenotaphrum secundatum* (Walt.) Kuntze), saltwater couch (*Sporobolus virginicus*), seashore paspalum (*Paspalum vaginatum* Swartz.), and kikuyu grass (*Pennisetum clandestinum*). All these grasses grow in harsh Australian environments—e.g., desert or coastal sites with low soil organic matter and nutrient status. Koronivia grass (*B. humidicola*) with strong BNI capacity was used as the positive control. The objectives of this study were to (1) screen the BNI capacity of Australian native or naturalised perennial grasses, (2) evaluate the interspecific variation of rhizosphere microbiota between studied perennial grasses, and (3) investigate the effect of BNI activities on the nitrifying microbes in the rhizosphere.

## 2. Materials and Methods

The perennial grasses used included the bermudagrass cultivar “Wintergreen”; St. Augustinegrass cultivar “Sir Walter”; native saltwater couch collected from South Australia (36.335° S, 139.754° E); native seashore paspalum collected from South Australia (35.544° S, 138.630° E); kikuyu grass cultivar “Whittet”; koronivia grass cultivar “Tully” sourced from the Australian Pastures Genebank (https://apg.pir.sa.gov.au/gringlobal).

The experiment was conducted in a growth chamber with a controlled environment (12 h of light at 800 µmol m^−2^ s^−1^ photosynthetic photon flux density and 30 °C, and 12 h of darkness at 20 °C). Autoclaved nutrient solution was applied, when required, with the baseline concentration as 22.2 mM CH_4_N_2_O (urea), 1.75 mM CaCl_2_, 0.16 mM MgSO_4_, 0.4 mM KH_2_PO_4_, 0.8 mM KC1, 125 µM H_3_BO_3_, 50 µM Fe-EDTA, 35 µM MnSO_4_, 3.0 µM ZnSO_4_, 1.25 µM CuSO_4_ and 0.75 µM H_2_MoO_4_ [25].

To remove the indigenous plant-associated microbiome, all the grasses were first grown from 2–3 cm stolons with nodes in pots (10 cm diameter × 20 cm tall) filled with sterilised sand and 50 mL of 1:10 diluted baseline nutrient solution was added every 3 days.

After 4 weeks, newly grown nodes were cut off and transplanted to pots (10 cm diameter × 20 cm tall) filled with field soil collected from a wheat field at Roseworthy campus, The University of Adelaide (34.537° S, 138.689° E). The soil is classified as a Calcisol, and the results of physicochemical analyses were pH (H_2_O): 6.97; EC: 0.093 dS·m^−1^; organic C: 1.45%; ammonium-N: 3.07 mg kg^−1^; nitrate-N: 14.0 mg·kg^−1^; Colwell P: 62.7 mg·kg^−1^; Colwell K: 653 mg·kg^−1^; clay: 25.5% and sand: 69.9%. Non-planted pots were used as controls for sampling bulk soil.

Pots were arranged as a randomised complete block design with 4 replicates in the same growth chamber with the same environment. Pots were watered by adding 30 mL of 1:10 diluted baseline nutrient solution and sterilised water to the field capacity every 3 days. After 8 weeks, plants were harvested for collection of rhizosphere soil. Firstly, soil > 5 mm away from the roots was carefully removed, and then roots with attached soil were gently shaken in sterilised bags to collect rhizosphere soil. Two grams of soil was subsampled and stored at −80 ℃ for DNA extraction, and the rest was used for BNI analysis.

BNI capacity was measured based on shaken soil slurries to test the potential nitrification rate (PNR). The method was modified from [9]. In brief, 15 g of soil was put into a 100-mL tube containing a combined solution of 1.5 mM NH_4_^+^ and 1 mM PO_4_^3−^ for incubation.

The combined solution was made by adding 15 mL (NH_4_)_2_SO_4_, 1.5 mL KH_2_PO_4_, 3.5 mL K_2_HPO_4_, and H_2_O to 1 L, and pH 7.2 was adjusted using dilute H_2_SO_4_ or NaOH. Tubes were shaken at 26 ℃ and 100 rpm. The slurry was sampled at 2, 4, 12, 24 and 48 h. NO_3_^−^ concentration was analysed using a Segmented Flow Analyzer (model AA1, SEAL Analytical, Mequon, WI, USA). The linear regression between measured NO_3_^−^ and the incubation time was analysed, and the slope was defined as the PNR. BNI capacity of each grass species was calculated as:

BNI capacity (%) = (PNR_bs_ − PNR_rh_)/PNR_bs_ × 100 (1)

where PNR_rh_ and PNR_bs_ are the PNR values of the rhizosphere soil of the tested grass, and the bulk soil in the same block, respectively.

Total DNA was extracted from 0.4 g of rhizosphere and bulk soil samples using the PowerSoil DNA isolation kit (MoBio, Carlsbad, CA, USA) based on the manufacturer’s instructions. Afterwards, the V3-V4 region of 16S rRNA genes with the forward primer 341F (CCTAYGGGRBGCASCAG) and reverse primer 806R (GGACTACNNGGGTATCTAAT) was amplified by PCR and sequenced on an Illumina MiSeq platform with 300 bp paired-end reads based on the manufacturer’s instructions.

The platform Quantitative Insights Into Microbial Ecology 2 (QIIME2) was used for bioinformatic analysis of the sequences [26]. De-multiplexing by the *q2-demux* plugin was conducted on the sequences, which were then trimmed and quality controlled using DADA2 [27] to generate an amplicon sequence variants (ASVs) table (*q2-dada2* plugin). The ASV was annotated against the SILVA reference database (vs. 132) [28] using the *q2-feature-classifier* plugin. All of the sequence data generated in the present study have been uploaded to National Center for Biotechnology Information with the project ID: PRJNA666294 and accessions ID: SAMN16287644.

The organellar ASVs (annotated as chloroplast or mitochondria) and less ubiquitous ASVs (present in <5% of samples) were removed from the ASV table. Variation of sequencing depth among samples was normalised using the Trimmed Mean of M values (TMM) method in the edgeR package in R [29]. Bray–Curtis distance between samples was calculated and used for the unconstrained principal coordinate analyses (PCoA) by the vegan package in R [30]. The ASV table was rarefied based on the minimum value of sequencing depth among samples, and the Shannon index was estimated as a measure of microbial diversity.

The effects of grass species on ASV composition were examined by performing the permutational multivariate analyses of variance (PERMANOVA) based on Bray–Curtis distance and 999 permutations (*adonis* function in the vegan package). The Statistical Analysis of Metagenomic Profiles (STAMP) [31] package was used to determine the effect of grass species on the relative abundance of microbial clades. The *p*-value was adjusted using the Benjamini–Hochberg FDR method in STAMP. ANOVA was applied to test the effect of grass species on BNI capacity and Shannon index by Minitab (Minitab Inc., State College, PA, USA).

## 3. Results

**Figure 1:**
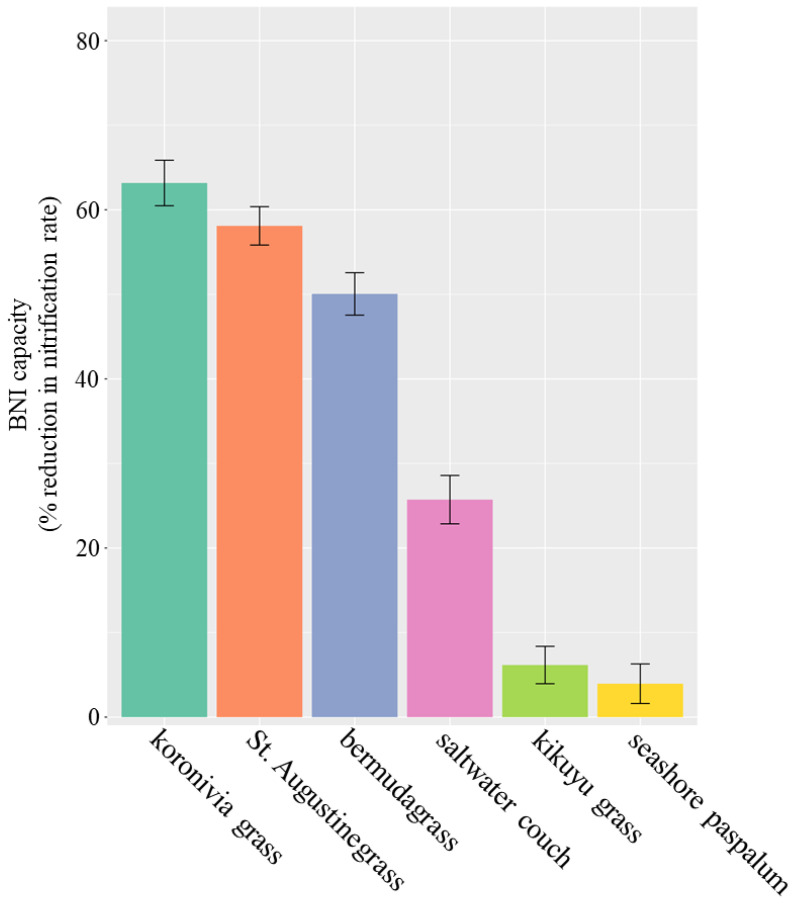
Biological nitrification inhibition (BNI) capacities of six perennial grasses. BNI capacity was measured by the reduction in potential nitrification rate in grass rhizosphere soil compared with the bulk soil. The error bar indicates standard error of four biological replicates at *p* = 0.05.

The genetic variation in BNI capacity among the six grass species studied here was significant (*p* < 0.01 in the ANOVA test) and large (Figure 1). The BNI capacity of three species—i.e., koronivia grass, St. Augustinegrass and bermudagrass—was about 10 times greater than those of kikuyu grass and seashore paspalum (50–60% vs. 4–6%). While koronivia grass had a BNI of 63%, St. Augustinegrass and bermudagrass also reached BNI values of 58 and 50%, respectively.

**Figure 2:**
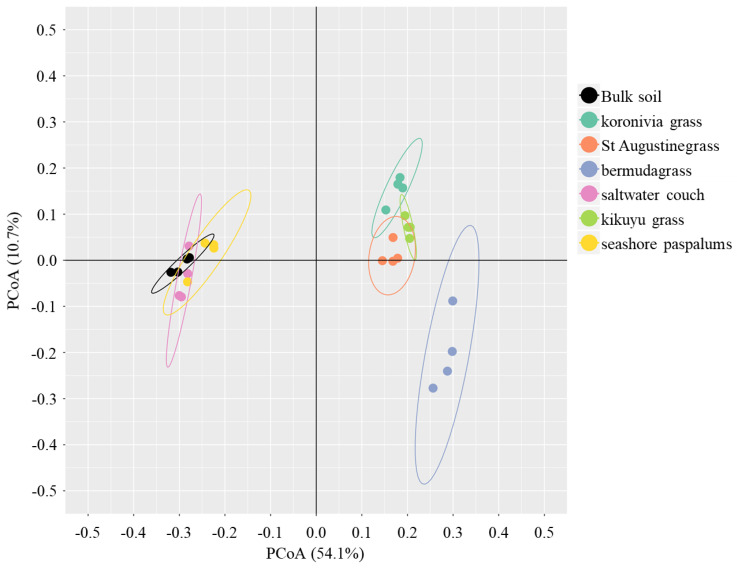
Microbiome composition in the bulk soil and rhizosphere soil of six perennial grasses. Unconstrained principal coordinate analyses (PCoAs) were performed based on the Bray–Curtis distance between samples using amplicon sequence variant (ASV) as the taxonomic unit. Ellipses indicate 95% confidence level to cover all the samples in each treatment.

Amplicon sequence variant was used as the taxonomic unit to compare the rhizosphere microbial composition of different grasses. The effect of grass species significantly drove ASVs’ composition (*p* < 0.01 in the PERMANOVA test, Table 1), and explained 44% of the variation with 32% variation from the block effect. The PCoA analysis showed a similar result—i.e., a large separation between grass species—and microbial ASVs’ composition in the rhizosphere of saltwater couch and seashore paspalum was closer to bulk soil, but differed from that of the other grasses (Figure 2).

**Figure 3:**
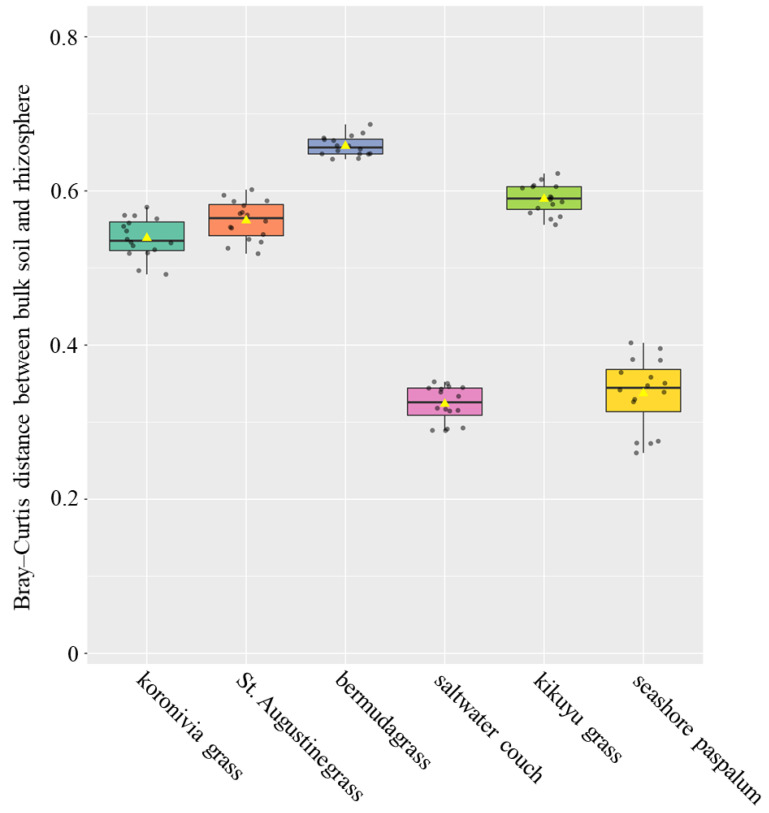
The differentiation of bacterial microbiome composition between bulk soil and rhizosphere soils of 6 perennial grasses. Bray–Curtis distance was used to indicate the difference in microbial ASVs’ composition between samples. In the boxplot, the values of individual samples are presented: the yellow triangle is the mean, and the box and central line represent first quartiles, medians, and third quartiles, respectively.

In addition, the Bray–Curtis distance for microbial ASVs’ composition was estimated between bulk soil and the rhizosphere (Figure 3). The distance was shorter in saltwater couch and seashore paspalum (*p* < 0.01, ANOVA test), indicating a high similarity between microbial communities in the rhizosphere and bulk soil, but was longer for bermudagrass—almost twice as large as for saltwater couch and seashore paspalum.

**Figure 4:**
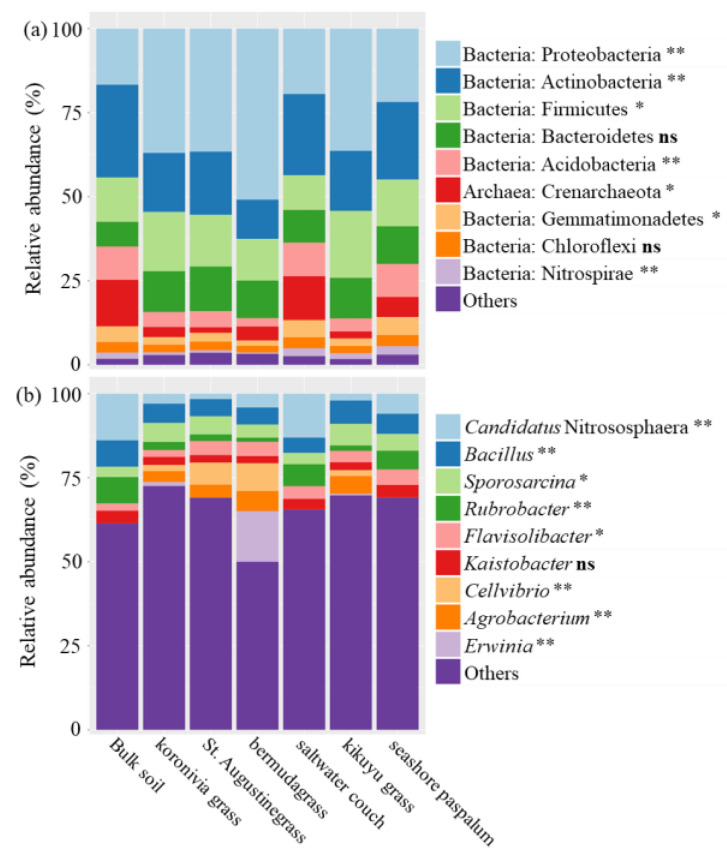
Taxonomic composition of bacterial communities in bulk soil and rhizosphere soil of six perennial grasses. Taxonomic resolution was at (**a**) phylum level and (**b**) genus level. The top 9 most abundant taxonomic groups are presented. The effect of treatment was tested as: ns: not significant, *: significant at *p* < 0.05, and **: significant at *p* < 0.01. *p*-values were adjusted by Benjamini–Hochberg FDR.

Consistent with the results of PCoA and Bray–Curtis distance analysis, there was a high similarity between bulk soil, saltwater couch rhizosphere and seashore paspalums rhizosphere in the taxonomic composition of microbiota, characterised by enriched Actinobacteria at the phylum level (Figure 4a) and *Rubrobacter* at the genus level (Figure 4b). For the other grass species (koronivia grass, St. Augustinegrass, bermudagrass and kikuyu grass), the dominant phylum in the rhizosphere was Proteobacteria, with a relative abundance of over 50% in bermudagrass (Figure 4a). At the genus level, *Agrobacterium* was barely detectable in the bulk soil and rhizosphere of saltwater couch and seashore paspalum with <0.04% abundance but represented around 4.6% in other grass species. St. Augustinegrass and bermudagrass harboured a greater proportion of rhizobacteria belonging to *Cellvibrio* (7.4 vs. 0.9%) than the others. Interestingly, *Erwinia* was the dominant genus in bermudagrass rhizobiome, accounting for 15.1% of the community, but was quite minor in the other grass species, at only 0.45% on average (Figure 4b).

Based on a review on nitrification [3], five microbial genera were considered as nitrifying microbes and their taxonomy and functioning enzymes are summarised in Table 2.

**Figure 5:**
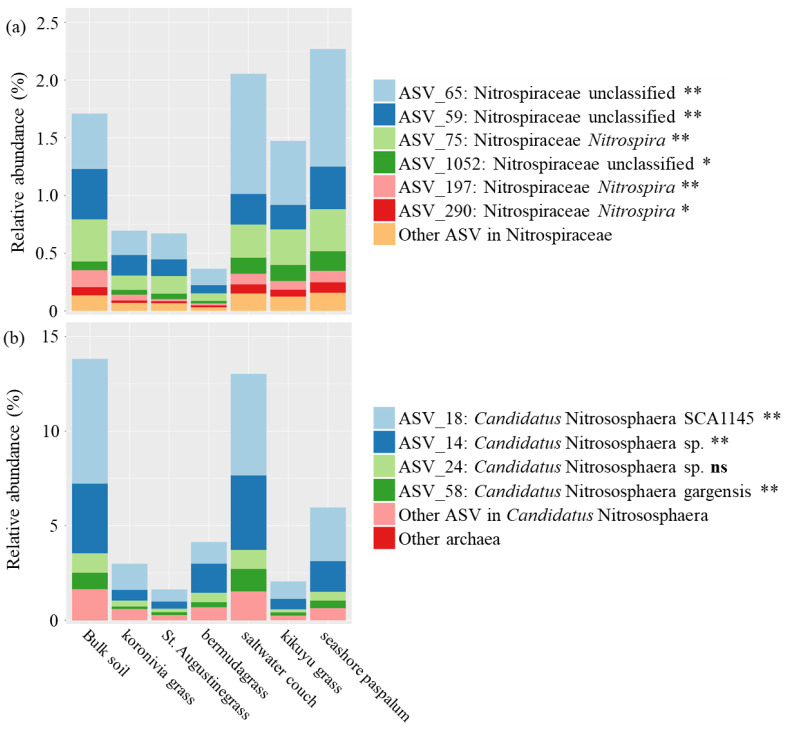
Relative abundance of potential ammonia-oxidizing (**a**) bacteria and (**b**) archaea in bulk soil and rhizosphere soil of six perennial grasses. The effect of treatment was tested as: ns: not significant, *: significant at *p* < 0.05, and **: significant at *p* < 0.01. *p*-values were adjusted by Benjamini–Hochberg FDR.

ASV annotated to the family Nitrospiraceae (including the nitrifying genus *Nitrospira*) accounted for 0.4–2.2% of the total microbial community in soils (Figure 5a). The relative abundance of six key ASVs (average relative abundance > 0.05% and in total accounting for over 80% of all ammonia-oxidizing bacteria (AOB)) is presented in Figure 5a. Three grasses with high BNI (koronivia grass, St. Augustinegrass, and bermudagrass) all harboured a decreased total abundance of *Nitrospira* (the genus including comammox bacteria and other nitrite-oxidizing bacteria (NOB), 0.58 vs. 1.93%) and decreased abundance of every identified key ASVs in family Nitrospiraceae in the rhizosphere, compared with bulk soil and rhizospheres of the other non-BNI grasses.

In contrast to the bacterial community, only four ASVs made up 85% of the archaeal community (Figure 5b). All of these four ASVs were annotated as *Candidatus* Nitrososphaera (an ammonia-oxidizing archaeal (AOA) genus, Table 2). Microbes assigned to *Candidatus* Nitrososphaera accounted for about 99.8% of the total archaea community in rhizospheres and bulk soil and had a higher relative abundance than the genus *Nitrospira* with nitrite-oxidizing function and potentially comammox bacteria, in the total microbial community (6.22 vs. 1.32% on average). For the grasses with strong BNI capacity, the relative abundance of ASV_18 assigned as *Candidatus* Nitrososphaera SCA1145 was significantly lower in the rhizosphere of high-BNI grasses (koronivia grass, St. Augustinegrass, and bermudagrass) and kikuyu grass than saltwater couch and seashore paspalum (1.02 vs. 4.10%), while ASV_14 (*Candidatus* Nitrososphaera sp.) was reduced in koronivia grass and St. Augustine grass. In addition, ASV_58 annotated as *Candidatus* Nitrososphaera gargensis was also less abundant in the rhizosphere microbiome of koronivia grass and St. Augustine grass.

The nitrifying genera *Nitrosomonas* and *Nitrosospira* (AOB, Table 2), belonging to the family Nitrosomonadaceae, were not detected in any samples in the present study. We did find two ASVs annotated to the family Nitrosomonadaceae, one unclassified Nitrosomonadaceae and one *Nitrosovibrio*, both of them accounting for a quite minor percentage of the microbiome (<0.05%).

No ASV was annotated to *Nitrobacter* (NOB, Table 2), another nitrifying bacterium belonging to the family Bradyrhizobiaceae. ASVs annotated as unclassified Bradyrhizobiaceae were detected but were not analysed here because most of the genera in the Bradyrhizobiaceae family have no nitrification functions.

The Shannon index of the microbial community was higher in bulk soil and the rhizosphere of saltwater couch and seashore paspalum (about 5.5–5.9, *p* < 0.01, ANOVA test), but was lowest in bermudagrass at only 4.7 (Table 3).

## 4. Discussion

### 4.1. BNI Capacity of Australian Perennial Grasses

Using perennial grasses naturalised in Australia, cultivars of St. Augustinegrass and bermudagrass had strong BNI capacities comparable to the koronivia grass cultivar “Tully”. “Tully” was previously ranked as having high activity in a BNI test of 18 plant species [5] and has been used as the benchmark to evaluate BNI performance [9,10]. Here, we confirmed the high BNI activity of St. Augustinegrass and bermudagrass which reached 80 to 90% of the level of BNI for koronivia grass. The N-use efficiency (estimated by biomass production under the same N conditions) of St. Augustinegrass and bermudagrass was greater than for seashore paspalum in previous observations [32]. This was probably associated with the large difference in BNI capacity between grass species identified in the present study, where seashore paspalum showed weak BNI activity of only 6% of that observed in koronivia grass (Figure 1).

In addition, an enormous variation in genetics, morphology, and tolerance to abiotic stresses has been observed among the ecotypes of Australian naturalised St. Augustinegrass [33] and bermudagrass [34,35]. In particular, there is a striking diversity of bermudagrass in Australia, and an Australian bermudagrass (potentially a native species) originating from an environment with low-fertility sandy soil and a hot, dry summer, a potential environment for the evolution of BNI, was characterised recently [36]. Hence, there appears to be a significant potential to discover Australian-adapted perennial grass ecotypes with stronger BNI activity than was found for the cultivars evaluated here. Previous findings confirmed a large intra-specific variation of BNI capacity within species [5,10,37]—e.g., compared with koronivia grass, higher BNI ability was detected in wheat landraces of *Triticum aestivum*, but not in modern wheat cultivars [10].

The BNI capacity measured in the present study was based on a soil assay that focused on the changes in nitrification potential and indicated the potential BNI capacity. Another method based on incubating bacteria in pure culture with root exudates has also been used to measure the actual BNI capacity. Both methods have shortcomings: our soil assay cannot directly measure the effect of root exudates in suppressing nitrifying microbes, and the incubation method involved collection of root exudates from hydroponic conditions and simplifying the complex soil microbiome, with only 1–2 nitrifying bacterial strains (e.g., *Nitrosomonas europaea* or *Nitrosospira multiformis*) being tested. The results from the two methods were correlated [9], or not [11], in previous studies. The advantage of the soil-based assay in the present research is that both the microbiome analysis and the BNI capacity analysis were conducted on the same soil samples, which enables their relationships to be revealed more accurately.

### 4.2. The Effect of BNI Capacity on Suppressing Potential Nitrifiers in the Rhizosphere Reflect

The relative abundance of identified *Nitrospira* spp. in the rhizosphere was reduced in all three grasses with high BNI activity. *Nitrospira*, a genus in Nitrospiraceae, has been identified with a nitrite-oxidizing function [38,39]. Certain *Nitrospira* species are “completely” nitrifying bacteria that are able to oxidise both steps of ammonia oxidation—i.e., NH_4_^+^ → NH_2_OH and NH_2_OH → NO_2_^−^ [40]. Furthermore, *Nitrospira* was reported to be widely prevalent in terrestrial ecosystems [41], and the growth of comammox *Nitrospira* was reduced by nitrification inhibitors in agricultural soils [42]. It has been reported that root exudates from koronivia grass significantly lowered the populations of pure cultured *Nitrospira* in in vitro testing [43], which is in line with our observations. More importantly, we demonstrated that a reduction in *Nitrospira* by BNI activity was based on the decreased relative abundance in the whole rhizosphere microbiome and occurred consistently in all the identified BNI grass species. As *Nitrospira* was the key nitrite-oxidizing microbe in the soil microbial community of the present study, and the relative abundance of other NOB (nitrite-oxidizing bacteria) was quite minor (e.g., *Nitrobacter*, Table 2), it is therefore possible that suppressing the nitrite-oxidizing step is the main mechanism for the high BNI grass species to inhibit nitrification. Further profile analysis of compounds present in root exudates is required to understand the detail act mode.

Almost all the archaea in the bulk soil and rhizosphere were classified as *Candidatus* Nitrososphaera, a potential ammonia-oxidizing archaea (AOA) genus. Unlike the response of *Nitrospira* to BNI activity, the reduction in different AOA populations was not fully induced by BNI activity. For example, a decrease in ASV_18 (*Candidatus* Nitrososphaera SCA1145) and ASV_58 (*Candidatus* Nitrososphaera gargensis) was induced not only by the high-BNI grasses but also the low-BNI kikuyu grass. A previous study on sorghum lines with diverse BNI capacity and under different N fertiliser regimes also showed that Nitrososphareaceae dominated the rhizosphere archaeal community, but their relative abundance was not associated with BNI activity [23]. Analysis of *amoA* gene copy numbers (genes controlling ammonia oxidisation) showed that the abundance of AOA was over 3000-fold more than AOB in soils from northern to southern Europe [44]. However, studies with DNA-based stable-isotope probes demonstrated that the main ammonia oxidation activities were from bacteria rather than archaea [45], which supports our result that populations of all detected potential comammox bacterial taxa were more sensitive to decreases due to BNI activity than AOA taxa.

AOB, *Nitrosomonas* and *Nitrosospira* (oxidizing ammonia to NH_2_OH), were not detected in the soil used in the present study (either bulk soil or rhizosphere soil). Previously, an assay for the suppression of *Nitrosomonas europaea* and *Nitrosospira multiformis* bacterial cultures by collected root exudates was developed to screen for BNI capacity of different plant species [5,14,21,22]. Our results reveal that a *Nitrosomonas-* and *Nitrosospira*-based BNI assay might not reflect the BNI capacity of plants, considering the complexity of the indigenous soil microbiome. Similar concern was also expressed by Coskun et al. [3], while here we provide supporting data to demonstrate the presence of *Nitrosomonas* and *Nitrosospira* were barely detectable populations in the plant-soil microbiome of BNI species. Previous studies found that *Nitrosospira* was highly abundant in the soil microbiome [46,47,48], possibly because those studies were in tropical regions with acidic soils, and low pH was identified as the key driver for the enrichment of *Nitrosospira* [49]. The soil in our study was from a semiarid Mediterranean environment with a neutral soil pH and our finding demonstrates that the comammox bacteria and AOA were the key players in soil nitrification.

### 4.3. Rhizospheric Selection for Microbiome Composition between Different Grass Species

The rhizosphere microbiome assemblages were determined by the different genera of grasses.

Two halophyte grass species, saltwater couch and seashore paspalums, were characterised by a weak selective power for rhizosphere bacteria from the bulk soil, indicated by the high similarity in bacterial composition and diversity between their rhizospheres and bulk soil (Figure 3 and Table 3). In extremely saline environments, halophytes have developed highly coevolved interactions with endophytes, the microbes inhabiting the interior of the root [50,51,52]. On the other hand, the rhizosphere selectivity for components of the soil microbiome might become weak when halophytes are grown in a mild environment as in the present experiment. Close correlations between soil and rhizosphere microbiomes of different halophytes have also been observed in previous studies [53,54]. For bermudagrass, St. Augustinegrass, and kikuyu grass, the rhizosphere microbiome structures were differentiated from the bulk soil, and also from each other, possibly due to the variation of root exudation activities in their rhizosphere. Studies profiling root exudates of wheat, chickpea and wild oat (*Avena barbata*) have demonstrated that the metabolite composition of the exudates was associated with the structure of the rhizosphere microbiome [17,55].

## 5. Conclusions

The present study demonstrated that Australian naturalised cultivars of St. Augustinegrass and bermudagrass were able to perform strong BNI, comparable with the BNI activity of the known BNI cultivar, *Brachiaria humidicola* “Tully”. BNI activity in perennial grasses suppressed the populations of *Nitrospira*, a genus with the nitrite-oxidizing activity including potential comammox bacteria, but not all of the potential ammonia-oxidizing archaea. The rhizospheres of two grass species, saltwater couch and seashore paspalum, exerted only a weak recruitment effect on the soil microbiome. Our results indicate directions for future research—e.g., evaluating genotypic variation in BNI capacity within the same perennial grass species collected from diverse Australian environments, identifying the active compounds released from the roots of the studied BNI grasses, and understanding the function and diversity of rhizosphere-nitrifying microbes that are sensitive to BNI activity.

## Figures and Tables

**Table 1:** The effects of grass species on rhizosphere microbiome composition. Permutational multivariate analyses of variance (PERMANOVA) analysis was performed using Bray–Curtis distance to indicate microbial composition. The block effect in the randomised complete block design was included and tested.

	*R* ^2^	*p*-Value
Block effect	0.32	<0.01
Grass species	0.44	<0.01

**Table 2:** The microbial groups with nitrification functions identified from previous summary [3]. Ammonia monooxygenase (AMO) catalysing NH_4_^+^ → NH_2_OH; hydroxylamine oxidoreductase (HAO) catalysing NH_2_OH → NO_2_^−^; nitrite oxidoreductase (NXR) catalysing NO_2_^−^ → NO_3_^−^. Comammox bacteria, complete ammonia oxidisers performing both ammonia oxidisation and nitrite oxidisation; ammonia-oxidizing archaea, AOA; ammonia-oxidizing bacteria, AOB; nitrite-oxidizing bacteria, NOB.

Taxonomic Genus	Nitrification Enzymes	Nitrifier Group	Results in Present Study
Bacteria; Nitrospirae; Nitrospira; Nitrospirales; Nitrospiraceae; *Nitrospira*	NXR	NOB	Figure 5a
Bacteria; Nitrospirae; Nitrospira; Nitrospirales; Nitrospiraceae; *Nitrospira;* certain species	AOM, HAO, and NXR	Comammox bacteria	Figure 5a
Archaea; Crenarchaeota; Thaumarchaeota; Nitrososphaerales; Nitrososphaeraceae; *Candidatus* Nitrososphaera	AOM and HAO	AOA	Figure 5b
Bacteria; Proteobacteria; Betaproteobacteria; Nitrosomonadales; Nitrosomonadaceae; *Nitrosomonas*	AOM and HAO	AOB	genus not detected
Bacteria; Proteobacteria; Betaproteobacteria; Nitrosomonadales; Nitrosomonadaceae; *Nitrosospira*	AOM and HAO	AOB	genus not detected
Bacteria; Proteobacteria; Alphaproteobacteria; Rhizobiales; Bradyrhizobiaceae; *Nitrobacter*	NXR	NOB	genus not detected

**Table 3:** Microbiome diversity in bulk soil and rhizosphere soils of perennial grasses. Diversity was indicated by Shannon index. Data are presented as mean ± standard error at *p* = 0.05. Within each column, means followed by the same letter were not significantly different, based on least significant difference (LSD) at *p* = 0.05.

	Microbial Community
Bulk soil	5.547 ± 0.062 ^b^
Rhizosphere soil:	
koronivia grass	5.446 ± 0.048 ^b^
St. Augustinegrass	5.463 ± 0.054 ^b^
bermudagrass	4.732 ± 0.123 ^c^
saltwater couch	5.769 ± 0.086 ^a^
kikuyu grass	5.374 ± 0.160 ^b^
seashore paspalums	5.925 ± 0.116 ^a^
